# Looking Backward, Looking Forward: The Long, Torturous Struggle with Mosquitoes

**DOI:** 10.3390/insects7040056

**Published:** 2016-10-19

**Authors:** Gordon M. Patterson

**Affiliations:** Florida Institute of Technology, 150 W. University Blvd., Melbourne, FL 32901, USA; patterso@fit.edu; Tel.: +1-321-674-7282

**Keywords:** mosquito control, history, emerging pathogens, medical entomology, environmental concerns

## Abstract

The American anti-mosquito movement grew out of the discovery of the role of mosquitoes in transferring pathogens and public concern about pest and nuisance mosquitoes in the late 1800s. In the 20th century, organized mosquito control in the United States passed through three eras: mechanical, chemical, and integrated mosquito control. Mosquito control in the 21st century faces the challenge of emerging pathogens, invasive mosquito species, and balancing concerns about the environment with effective control strategies.

This review is drawn from Gordon M. Patterson’s Mosquito Wars (University Presses of Florida 2004) [1] and The Mosquito Crusades (Rutgers University Press 2009) [2].

## 1. Introduction

In October 1770, Johann Wolfgang von Goethe, who would later become Germany’s most famous poet, writer and statesman, made an excursion to a village on the Rhine called Sesenheim. There, the 21-year-old von Goethe, who was studying law in nearby Strassbourg, met and fell in love with Frederike Brion, the daughter of the town’s Protestant minister. A brief, intense love affair ensued. One afternoon during an excursion a brood of “entsetzichen Rheinschnaken” (horrible Rhine mosquitoes) drove the young lovers back to Sesenheim. Later, von Goethe declared to Frederike’s father that the existence of mosquitoes made him doubt God’s goodness. How, von Goethe wondered, could an “omniscient, omnipotent, and benevolent” Deity have blighted creation with mosquitoes? The virtuous parson disagreed explaining that in the Garden of Eden mosquitoes were gentle, pacific creatures. It was only “after the fall of our first parents” that mosquitoes came to prey on human beings. Von Goethe retorted that it surely must have been mosquitoes and not an “angel with a flaming sword” that had driven “the sinful couple” from paradise [3] (p. 533).

Von Goethe was unaware that, at that very moment, a revolution in human understanding of the insect world in general and mosquitoes in particular was underway. A century before von Goethe’s musings, Robert Hooke (1665) offered a detailed description of mosquitoes in his *Micrographia, or Some Physiological Descriptions of Minute Bodies made by Magnifying Glasses.* Improvements in the compound microscope which was invented in the 16th century gave Hooke, John Swammerdamm, and most notably Jean Rèaumur unprecedented insight into the world of insects. In 1738, Rèaumur devoted his fourth memoir, *Histoire des Cousins*, to describing the life history of *Culex pipiens.* Unlike von Goethe, Rèaumur believed that no part of God’s creation was unworthy of admiration. Today, Rèaumur would be called an ecologist. He believed that each living thing is connected to all of the other living things that surrounded it. Rèaumur opened a new chapter in the history of entomology. His accounts of the life histories of insects are striking for their detail and clarity. Nowhere is this more clearly shown than in his discussion of mosquitoes. Rèaumur wrote:

“I have received written observations on various insects, and particularly on mosquitoes, from a learned Chartreux [monk] who amuses himself and busies himself admiring the works of the Almighty wherever he isn’t occupied in singing His praises. He has studied mosquitoes more and longer than the authors who have written about them. Not only has he found that their worms leave skins behind them but he is satisfied that they leave three of them aside from the last to which are followed by changes in the insect’s form. If all these pious monks—had a penchant for observing insects, we might expect that the most essential points in the life history of these little animals would soon be made known. What diversion could these monks propose more worthy of the life they have embraced than the one which would place under their eyes the marvelous creations of a Power unlimited? For therein, even their leisure would give them more occasions to adore Him than have those [other monks] who are diverted from Him by too many occupations, whether serious or frivolous [4] (p. 8).”

Reading Rèaumur’s *Memoires pour servir a l’Histoire des Insectes* provides insight into his fascination with mosquito anatomy and physiology. Most striking is the mixture of respect and admiration with which he speaks of mosquitoes. He wrote:

“Mosquitoes are certainly our declared enemies, and very annoying enemies at that. But they are enemies good for us to know. If we give them even the least bit of attention we find ourselves forced to admire them and to admire even the instrument they bleed us with; for this it is only necessary to examine their structure. Furthermore, throughout their lives they offer facts adequate to please any of those minds which are curious about the marvels of nature. There are even times in the mosquitoes’ lives when, having made the observer forget that they’ll persecute him some day, they make him feel actually, solicitous about their welfare” [4] (p. 7).

Few have written with greater eloquence and sensitivity about mosquitoes.

Rèaumur’s sympathy for mosquitoes, however, did have limits. Like von Goethe, he reported that he encountered peasants in the country side with such severe mosquito bites on their arms and legs that he feared amputation might be necessary. Horror stories about mosquitoes in the 18th and early 19th century abounded. In 1736, the people of Salisbury, England, were terrified by “vast columns” of mosquitoes which darkened the skies above the city’s famous cathedral. Many mistook the brood of mosquitoes for smoke and thought that the cathedral was on fire. Twenty years later another sinister “black cloud” of mosquitoes descended on Oxford…almost totally intercepting the beams of light” [5] (pp. 115–116).

In 1758 Carl Linnaeus applied his binomial classification system to mosquitoes in the tenth edition of his *System of Nature* [Reference number]. By 1900 taxonomists such as Johan Fabricius, Johann Meigen, Frederic Skuse, Daniel Coquillett and others had identified more than 160 species of mosquitoes [6] (p. 9). Throughout this period most people regarded mosquitoes as being like the weather. They could be complained about but ultimately nothing could be done about them.

The discovery in the closing decades of the 19th century that mosquitoes could transmit pathogens opened a new chapter in the story of the relationship between mosquitoes and human beings. Until 1871 no medical textbook identified any disease that could be transmitted by an insect [7] (p. 565). During the next 30 years, Patrick Manson (filariasis), Carlos Finlay (yellow fever), Roland Ross (malaria), Giovanni Grassi (malaria), the Reed Commission (yellow fever), and Harris Graham (dengue) identified the role of mosquitoes as pathogens vectors. During the first decade of the twentieth century, the pioneering campaigns of William Gorgas in Havana and Panama, Oswaldo Cruz in Brazil, Malcolm Watson in the Federated Malay States demonstrated the efficacy of mosquito control in reducing mosquito-borne pathogens. Simultaneously, the call for mosquito control gained impetus in citizen groups seeking relief from nuisance mosquitoes.

The American anti-mosquito movement moved through three distinct phases in the twentieth century: a mechanical control era 1900–1942; a chemical control era 1942–1972; and, an integrated mosquito management era 1972–present. As the second decade of the 21st century nears its conclusion, advocates of mosquito control face a daunting array of challenges ranging from emerging pathogens and invasive species to the lack of support for research and opposition from increasingly vocal critics who believe mosquito control is in conflict with protecting the environment.

## 2. The Mechanical Era 1900–1942

The mechanical era of mosquito control drew its impetus from the Gorgas’s success in Havana and his later work in Panama. In 1901, a group of well-to-do residents of South Orange, New Jersey formed a village improvement society motivated by the desire to reduce the mosquito menace. Spencer Miller, president of the South Orange Improvement Society, enlisted the support of Leland Osian Howard, the head of the Bureau of Entomology at the United States Department of Agriculture (USDA), and John Smith, a professor of entomology at Rutgers University. Four years later, Smith published his ground breaking *Mosquitoes of New Jersey* which outlined a scientifically based campaign against mosquitoes based on ditching and draining and the limited use of oil as larvicide [8]. In 1912, Smith and his allies won the support of Woodrow Wilson, New Jersey’s governor, for the passage of the first law authorizing the creation of mosquito abatement districts.

Smith’s work proved a model for advocates of mosquito control in California in 1905. Citizens in Burlingame and San Mateo requested help from the entomology department at the University of California in finding relief from salt marsh mosquitoes. Four years later, a young Ohio entomologist named William Herms joined the Berkeley faculty. Herms had little interest in pest and nuisance mosquitoes. In 1910, he began California’s first campaign to control anopheline mosquitoes in Penryn, California. A year later he sought to win state approval for an abatement act to control pathogen-bearing mosquitos. Herms failed twice (1911 and 1913) in his effort to secure a law allowing counties to organize mosquito control districts. In 1915 California authorized the formation of abatement districts when Herms won the support of the powerful real estate lobby which feared mosquitoes in certain areas would adversely affect property values.

During the next 20 years, mosquito control programs based on ditching and draining with the limited application of oil were launched in a number of states such as Florida, Utah, and Illinois. Florida’s anti-mosquito campaign bears particular note because advocates of mosquito control built their campaign around the effort to control pathogen bearing mosquitoes. Florida had long been plagued with outbreaks of yellow fever and dengue fever. In the summer of 1922 a dengue epidemic swept across the Gulf Coast and Sunshine State [9]. Miami, which had instituted an *Ae. aegypti* control program, was spared. In December 1922, 100 proponents for mosquito control met in Daytona, Florida, and formed the Florida Anti-Mosquito Association. They chose Joseph Porter, who had served as the state’s first health officer, as their inaugural president. The closing words of Porter’s presidential address were to become the mantra for mosquito control workers in Florida and across the nation. Porter declared, “Keep everlastingly at it” [10] (p. 3).

Notable advances in mosquito control took place in the 1920s. The United States Department of Agriculture (USDA) established an experimental station on mosquitoes in Mound, Louisiana. Under the direction of Willard King, USDA researchers explored different means of controlling mosquitoes. In 1925, King and George Bradley performed the first test using an airplane to distribute Paris Green, an emerald green arsenite, as a larvicide against anopheline mosquitoes. At the same time, United States Public Health Service (USPHS) entomologists continued their research on different species of mosquitos’ flight ranges and different strategies for eliminating malaria in the Southeast. In 1927, Congress approved funding for a survey of Gulf and Atlantic Coast salt marsh mosquitoes. Thomas Griffitts, a USPHS officer led the two-year project. In 1929 he summarized his findings at the meeting of the Gulf and South Atlantic Anti-Mosquito Congress. Griffitts judged the new group a “wide awake organization” and expressed optimism that with determination and sustained effort 5,600,000 acres along the coasts could be won back from salt marsh mosquitoes [11] (p. 151).

The Great Depression provoked tremendous changes within the anti-mosquito movement. The economic downturn forced the leaders of the anti-mosquito movement to temper their optimism and reconsider their objectives. Prospects for mosquito control dimmed as the depression worsened. The ambitious plans for the Gulf and Atlantic Coast Anti-Mosquito Association were shelved. The anti-mosquito movement reached its nadir point in 1933. Four years of depression robbed communities of the resources and political will to support the mosquito crusade.

In April 1933, Fred Bishopp, Chief of the USDA’s Division of Insects Affecting Man and Animals, suggested “that the great public benefits to be derived from anti-mosquito operations and the excellent opportunity which it gives for utilizing large numbers of the unemployed cannot be too strongly emphasized” [12] (p. 80). Massachusetts provided an example of how other states and the federal government could use mosquito control to provide jobs for the then large numbers of the unemployed. In Massachusetts, the State Reclamation Board’s “first concerted action was on the island of Nantucket.” In 1930, the legislature provided support to the Cape Cod Mosquito Project as part of the state’s depression relief initiative [13] (p. 80).

Franklin Roosevelt’s New Deal buoyed Bishopp’s hopes for sustaining mosquito control. During Roosevelt’s first 100 days in office the President set in motion a chain of events that were to have a profound effect on the mosquito crusade. Mosquito control became an integral element in both the Tennessee Valley Authority (TVA) and the federal work relief initiatives that grew out of the Federal Emergency Relief Administration (FERA) and its sister agency, the Civilian Works Administration (CWA). These programs present an impressive display of the strengths and weaknesses of the mechanical era of mosquito crusade.

Mosquito control was to become an integral component in the TVA from the project’s beginning. When Roosevelt signed the bill authorizing the TVA, there was widespread awareness of threat from malaria posed by the mosquitoes from impounded waters. Fortunately, there was also a substantial body of research demonstrating that certain water management strategies could significantly reduce this threat. USPHS research demonstrated that mosquito control could significantly reduce the risk of malaria outbreaks.

Edward Bishop was then leading the TVA’s Health and Sanitation Section. Bishop, who served as Tennessee’s state health officer, had extensive experience with malaria in the rural South. By November 1933, Bishop had prepared “plans for a comprehensive malaria control program” [14] (p. 302). Drawing on Henry Rose Carter, Thomas Griffitts, and Joseph Le Prince’s studies, Bishop formulated a “naturalistic control” strategy for the TVA. Bishop recognized that there was no single remedy for malaria. The USPHS researchers had established in their demonstration projects at Roanoke Rapids, Electric Mills, and in Arkansas that it was possible to eliminate malaria through a combination of strategies. Permanent shoreline improvements, fluctuations (seasonal and periodic) of the water level in impounded sections, use *of Gambusia affinis* to prey on mosquito larvae, and limited larviciding offered a means to reduce anophelines. Screening houses and providing quinine to those living near the reservoirs offered an additional line of defense against malaria bearing mosquitoes. Bishop argued that each impoundment was unique. The key was to remain flexible and employ a mosquito control plan that was tailored to each site’s ecology.

The Public Works Administration (PWA) under FERA provided loans for construction projects under Roosevelt’s New Deal programs. To facilitate immediate construction projects the New Deal created the Civilian Works Administration (CWA). This represented a fundamental change in the federal relief effort. The CWA would for the first time make the federal government responsible for finding work for unemployed Americans.

Mosquito control benefited from the CWA activities in 1933/1934. President Roosevelt put Harry Hopkins in charge of the CWA. Hopkins asked the USDA, the USPHS, and a handful of other federal agencies for a list of projects that could provide immediate employment. Bishopp argued that mosquito control could provide jobs for hundreds of thousands of workers. Hopkins agreed and asked Bishopp and Louis Williams, Jr. to supervise the CWA mosquito control projects.

The USPHS intensive malaria drainage project was one of four CWA health initiatives. The other projects included: the construction of sanitary privies; typhus surveys; and, sealing abandoned mines. Hopkins allocated $4.5 million dollars of CWA funds for the southern mosquito control work. Ditching operations began in early December and by 20 January 1934, 28,000 men were at work. Supplemental FERA grants to southern states allowed an additional 53,000 men to join the ditching effort. At its high point, USPHS officials estimated that 120,000 workers were digging mosquito drainage ditches in the South [15] (pp. 2–3).

Unfortunately, there was little time to plan the work, a shortage of experienced personnel further reduced effectiveness. “On such short notice”, Louis Williams, who supervised much of the CWA work, conceded, “it was not possible to organize technical supervisory forces in 14 states” [16] (p. 11). This meant that USPHS supervisors had to rely on the local officials to plan the drainage and provide competent managers to oversee the work. Many local authorities placed their highest priority on ensuring paychecks for the unemployed and not providing mosquito control. There were also numerous disputes between local and federal officials over their different responsibilities for administrating the work and things like providing tools, boots, and transportation for the workers.

Louva Lenert, who served as a consultant for the Rockefeller Foundation, warned that without regular maintenance, the CWA malaria ditching would have little effect on the malaria rate. In 1934, Lenert offered a somewhat pessimistic assessment of the federal program. He observed, “…the [CWA] drainage program alone is not expected to have great permanent value, however, unless it is followed by continued maintenance” [17] (p. 80). 

The CWA pest mosquito project presented an even more daunting challenge. Like Williams, Fred Bishopp was under orders to get men to work on mosquito control projects immediately. The malaria work was limited to 14 southern states where the USPHS had extensive experience since 1912. The CWA pest work stretched from New York to California. Many states lacked any experience with mosquito control. Bishopp, however, remained optimistic. He was adamant that “mosquito control work is one activity which has been promoted rather than handicapped by the depression” [18] (p. 37).

In 1937 the Audubon Society attacked mosquito control. “The zeal for drainage”, WilliamVogt wrote, “has ravaged the North American continent like some form of terrestrial erysipelas. Wildlife and vegetation have been killed, and the earth has dried up”. The federal emergency support of malaria and pest mosquito control ditching was but the most recent assault on the nation’s water resources. Vogt argued that the federal mosquito control work was a “publicly financed sales talk…that under the guise of ‘education’ officials concerned with mosquito control have made sweeping claims without producing any scientific proof”. Citing the work of Clarence Cottam, Warren Bourne, and F.M. Uhler at the USDA’s Biological Survey, Vogt charged the CWA, CCC, and WPA sponsored malaria and pest mosquito control initiative was no more than a “government-sponsored racket” [19] (pp. 7 and 15).

The dispute reached a boiling point when the USDA’s Biological Survey unit blocked a pest mosquito control project in Rhode Island. Milton H. Price, supervisor of Rhode Island’s pest mosquito work, reported that early in 1937 all of the projects he had submitted to the WPA were returned “with the notation, ‘disapproved by the United States Bureau of Biological Survey’”. The work remained stalled until Cottam and Bertrand Smith visited Rhode Island and reviewed the proposal. Cottam and Smith “approved the projects, with reservations, and within two weeks”, Price told an Atlantic City audience of mosquito control proponents, “we were able to begin work on projects that had previously been disapproved”. The precedence had been set where wildlife advocates succeeded in temporarily blocking and modifying a mosquito control project [20] (p. 112).

The CWA, CCC, and WPA mosquito projects during the mechanical era of mosquito control created a legacy of hostility between advocates of mosquito control and an increasingly vocal group of hunters, fishermen, and wildlife advocates. The Third Annual National Wildlife Conference held in Baltimore in March 1938 revealed the depth of the distrust between mosquito control proponents and the wildlife advocates. J. “Ding” Darling had organized the first National Wildlife Conference in 1936. The Third Conference in 1938 devoted a special session to a debate over the question: “What is Wrong with Mosquito Control?” Clarence Cottam and William Vogt presented the case against mosquito control. Fred Bishopp and Louis Williams, Jr. delivered the mosquito control proponent rebuttal [21] (pp. 81–107).

The Third North American Wildlife Conference marked the symbolic close to the first phase of the mosquito crusade. The era that began with John Smith’s call for ditching the New Jersey salt marshes came to its close in the late 1930s. The first generation of mosquito control professionals relied on shovels, ditching machines, and oil in their fight against mosquitoes. In the 1940s, a new generation of mosquito control professional would improvise new tools that opened new possibilities for mosquito control in the “chemical era”.

## 3. The Chemical Era

The anti-mosquito movement underwent radical changes in the 1940s and 1950s. Concerns about wildlife and environmental issues faded as war clouds gathered over the Pacific and in Europe. New insect-borne diseases emerged to threaten both civilians and the armed forces. After Pearl Harbor, experienced mosquito control workers like William Herms, Stan Freeborn, Fred Bishopp, and Louis Williams led the effort to protect soldiers, sailors, marines, and war workers from insect-borne pathogens. In laboratories in Orlando, Florida and Beltsville, Maryland, USDA researchers pioneered innovative means of chemical control. Simultaneously, scientists and physicians at the Rockefeller Foundation and engineers and entomologists at the TVA explored new strategies to reduce malaria parasites and control disease vectors. Finally, as Allied Forces were advancing, Robert Glasgow, New York’s state entomologist, Don Rees, a professor in Utah, Thomas Headlee, New Jersey’s state entomologist, and Tommy Mulhern, a veteran New Jersey mosquito control professional, organized American Mosquito Control Association with the goal of catalyzing the expansion of mosquito control in the post war era.

During World War II protecting recruits and inductees from mosquito-borne diseases posed a formidable challenge. Despite the USPHS’s efforts during World War I, there had been more than 10,000 cases of malaria in the military [22] (p. 27). Dr. Joseph Mountin, director of the USPHS’s State Service Division in Washington, made establishing a comprehensive malaria control program a priority. In 1940, Mountin sent Louis Williams to serve as the USPHS’s liaison with the Fourth Army Corps headquarters in Atlanta [23] (p. 92). Williams, who began his career in malaria control in World War I, had recently returned to America from Southeast Asia where he had served as chief of the Malaria Commission for the China Burma Highway project. His experiences in Asia strengthened his conviction in the importance of malaria control for the war effort.

In 1941, the Secretary of Agriculture placed Fred Bishopp in charge of the USDA’s wartime research efforts. Initially, Bishopp asked Willard King to oversee the insecticide and repellent research at the USDA’s Orlando laboratory. King was called to active duty in the army sanitary corps before the work started. In July, Bishopp chose Edward Knipling to lead the research team.

The story of the Orlando laboratory’s accomplishments is remarkable. During the next 36 months, USDA entomologists, toxicologists, chemists and engineers tested more than 10,000 chemical compounds. Initially, the primary objective of the lab was to conduct investigations in the control of body lice (the vector of typhus) and insect repellents for mosquitoes, biting flies, mites, fleas, ticks and bedbugs. Knipling, the laboratory’s director, told the researchers that they were not to concern themselves with basic research. Their objective was to examine and quickly assess the value of various materials that might be of use against pathogen-bearing insects and pests.

The goal was to find practical tools that could be used in combat. “We cut corners”, Knipling recalled, “whenever possible to reach an end point and to make specific recommendations or to suggest what might be tried by the army in large-scale practical tests” [24] (p. 33). Fred Bishopp emphasized the need for speed. “Disease”, he explained, “was a more formidable enemy than the Japs and that without the effective control of disease the outlook for early defeat of the Japanese was indeed gloomy” [25] (p. 376).

Late in 1942, the Orlando researchers made a revolutionary breakthrough. In November, Bishopp forwarded samples of an insecticide he had received from the Geigy Chemical Company of New York. The product, called Gesarol, had been developed in Geigy’s Swiss laboratories as a potential tool against moths and lice. In Orlando, USDA chemists identified the active agent in Gesarol as the chemical compound dichloro-diphenhyl-trichloroethane. Knipling’s researchers followed the lead of British chemists and used the initials DDT (dichlorodiphenyltrichloroethane) to designate the chemical agent.

The discovery of the insecticidal properties of DDT marked the beginning of a new epoch in mosquito control. Since John Smith’s pioneering work in the New Jersey meadowlands and marshes, ditching and drainage had served as the principal strategy for mosquito control. Chemical means of control were limited to oil and, after 1921, Paris green. Both were effective only as larvicides. In the early 1930s, Joseph Ginsburg’s research on the toxicity of pyrethrum flowers led to the development of a new product known as the New Jersey Mosquito Larvicide. DDT’s low cost, potency, persistence, and efficacy as both a larvicide and adulticide revolutionized mosquito control.

Field tests in early 1943 confirmed DDT’s potency against lice, mosquito larvae, and adult mosquitoes. In May 1943, Neocide, the Orlando laboratory’s formulation of a DDT louse powder, was added to the list of approved pesticides. The first shipments of Neocide reached North Africa in the late summer. Fred Soper, a Rockefeller Foundation scientist who had relied on Paris green in the 1930s to eradicate *Anopheles gambiae* in Brazil [26] (p. 135), was one of the first to use Neocide in delousing German prisoners in North Africa. Neocide’s power lay in its persistence. Other insecticides required repeated treatments. A single application of Neocide lasted for months.

DDT’s critical test came six months later in Naples, Italy. Typhus reached epidemic proportions. The victorious allied forces succeeded in liberating the city only to find themselves caught in a battle against an epidemic that threatened both civilians and soldiers. Between December 1943 and March 1944, Soper and his colleagues deloused more than two-and-a-half million people in southern Italy. At the height of the anti-typhus campaign in Naples “the army dusted fifty thousand people per day with DDT” [26] (p. 145). The Orlando DDT louse powder had a miraculous effect. “For the first time in the history of medical science”, a contemporary observer noted, “a typhus epidemic was stopped in mid-winter” [27] (pp. 63 and 68).

The discovery of DDT’s efficacy sparked a dramatic expansion of the USDA Orlando laboratory’s mission. The laboratory’s initial funding from the Office of Scientific Research and Development was targeted on the limited objectives of devising louse control measures and developing new repellents. In July 1943, Knipling redefined the laboratory’s mission. He divided the researchers into four teams. Four of the laboratory’s 19 workers would continue work on lice. Knipling assigned the remaining entomologists, chemists, and technicians into three teams focusing on mosquito larvicides, adulticides and insect repellents.

Entomologists at the TVA contributed to the research on DDT’s effectiveness as a larvicide and adulticide. In June 1943 at Wilson Dam in Alabama, DDT was sprayed for the first time from an airplane [28] (p. 77) (Other aerial tests followed against larvae and adult mosquitoes at the Banana River Naval Air Station near Cocoa, Florida. In Stuttgart, Arkansas, USDA researchers demonstrated DDT’s phenomenal residual power in a series of tests in 1943 and 1944. Finally, in May 1945, the Orlando laboratory and the Rockefeller Foundation launched a major experiment in Mexico. The researchers treated a group of villages with high malaria indexes with DDT. Four months after the application there was nearly a complete eradication of adult mosquitoes within the treated buildings. The mosquito larvae in rice fields, which were adjacent to the village, dropped 90% [29] (p. 41).

Several entomologists expressed reservations about DDT. At Rutgers, Joe Ginsburg called for restraint. Ginsburg, who developed some of the first mosquito insecticides, warned of DDT’s potential harm to wildlife. In July 1943, Knipling sent Ginsburg a sample of DDT for testing. Ginsburg conducted 32 field experiments using DDT as a larvicide in Essex, Middlesex, Monmouth, Passaic, and Union counties in New Jersey. He reported that “DDT, in concentrations sufficiently high to kill subsurface feeding larvae, was toxic to at least three species of fish”. Ginsburg’s conclusion was that “DDT may offer a new and highly potent weapon to supplement our present chemicals used in the control of mosquitoes. But before it can be recommended for practical use, more extensive field investigations under various mosquito breeding conditions are necessary” [30] (pp. 53–54). 

While Ginsburg was raising concern about DDT’s effect on wildlife, Philip Granett, also at Rutgers, provided the Orlando laboratory with protocols for its research on insect repellents. In 1935, Granett began research on finding a repellent that would be effective against *Ae. aegypti* mosquitoes. Granett tested more than 1000 different compounds. By 1940, Granett had identified three promising candidates: Dimethhyl phthalate, Sta-Way, and Rutger’s 6–12 [31] (p. 38).

The Orlando group adopted Granett’s approach to testing repellents. Instead of focusing on *Ae. aegypti*, the Florida researchers focused on finding a repellent against the most important southern vector of malaria, *An. quadrimaculatus* [32] (p. 187). The challenge facing the Orlando researchers was to formulate a repellent that would be effective across a wide-range of insect pests. Eight thousand different formulations were tested. A mixture consisting of 60% of Granett’s dimethhyl phthalate, 20% Indalone, and 20% Rutgers 6–12 proved most effective [33] (p. 53). During the war years, the military purchased more than “one hundred and fifty million two-ounce bottles of synthetic organic repellents” [32] (p. 186).

Neither 6-2-2 nor any other of the repellents developed in Orlando or Rutgers were panaceas. At best, the repellents provided limited protection against only a few mosquito species. For most soldiers the compelling argument against the repellents was that they were unpleasant to wear. “Unless mosquitoes were biting in unendurable numbers”, Emory Cushing concluded, “the soldiers were disinclined to use repellents. Some combat troops refused to use them on the grounds that in the humid areas the odor was detectable by the enemy” [34] (pp. 48–49). It was, they decided, far better to be bitten by a mosquito than killed by Japanese infantryman. The search for an effective repellent continued in Orlando after the war’s end. In 1954, USDA researchers in Orlando succeeded in formulating a broad spectrum repellent *N,N*-diethyl-m-toluamide called DEET.

Fueled by the availability of DDT and, later, by the subsequent discovery of other powerful chemical toxicants, the anti-mosquito movement experienced spectacular growth during the two decades following World War II. The chemical era of mosquito control was marked by an explosion of new mosquito control programs from the Gulf Coast to the Pacific Northwest. New state and regional anti-mosquito associations formed in Virginia, Utah, Texas, Louisiana, Oregon, and Washington. The greatest growth took place in California and Florida. By the beginning of the 1960s, California and Florida’s anti-mosquito programs towered over the rest of the nation both in the size of their operations and their commitment to research in mosquito control.

In 1962, Rachel Carson gave voice to the unease of individuals concerned about the environmental impact of the use of insecticides. Carson’s *Silent Spring* exposed a growing discontent with mosquito control’s reliance on pesticides. On the first Earth Day, 22 April 1970, protesters marched in Chicago, Boston, San Francisco, Los Angeles, Fort Lauderdale and hundreds of other American cities calling for a ban on DDT and stringent restrictions on mosquito control programs.

There is a near tragic quality in the development of the mosquito control movement in the decades following World War II. The use of DDT and reliance on chemical control agents which sparked the spectacular growth of mosquito control in the post-War era became the source of the public’s disenchantment with mosquito control in the turbulent 1960s. The remarkable thing is that at the beginning of the insecticide era, many of the leaders of the mosquito crusade cautioned against an over reliance on DDT [35] (pp. 27–29).

The late 1960s brought a tectonic shift in the mosquito and vector control movement. For 70 years, the advocates of mosquito control had perceived themselves as champions of public health and the first line of defense against the torment of winged, blood-sucking pests. The publication of Carson’s *Silent Spring* in 1962 signaled the beginning of a new era in American concerns about the environment. One year later Congress approved an appropriation for American participation in the Pan American Health Organization’s (PAHO) *Ae. aegypti* eradication initiative. In 1947, the U.S. had joined with other PAHO states in calling for the elimination of *Ae. aegypti* in the Western Hemisphere. Sixteen years elapsed before the U.S. participated in the campaign. Difficulties were present from the start. Elizabeth Etheridge contends in her history of the Centers for Disease Control (CDC) that U.S. public health workers fought against the eradication bill “tooth and nail”. CDC officials argued that there were more important public health needs. The last U.S. yellow fever outbreak had taken place in 1905. There had not been a single case of yellow fever in the United States since 1925. “Hardly anyone [at the CDC]”, Eldridge argued, “saw the eradication of the *Aedes aegypti* mosquito…as a pressing health concern”. The CDC’s leadership believed that “the decision to eradicate the *Aedes aegypti* mosquito was a political one, a matter of foreign policy [23] (p. 122)”.

The U.S. program was underfunded from the start. Moreover, the program’s reliance on DDT and other pesticides placed it in the center of the growing controversy about DDT. By 1968, the political will in the U.S. necessary to sustain the eradication program had vanished. An embattled Congress confronted with funding the unpopular War in Vietnam and the Johnson Administration’s War on Poverty terminated the *Ae. aegypti* eradication program.

*Silent Spring*’s publication and the debacle of the *Ae. aegypti* Eradication Program added to the public’s disillusionment with mosquito control. In 1972, the newly created Environmental Protection Agency (EPA) banned DDT’s use in the United States. Veteran mosquito professionals were in a new world. The public’s faith in the promises that had launched the chemical era had come to an end. In the coming decades mosquito control would face a myriad array of new challenges.

## 4. The Integrated Mosquito Management Era 1972 to Present

While the debate about the use of DDT continued a handful of veteran mosquito control workers believed that a “the third age” of mosquito control was dawning. “The future can be bright”, John Mulrennan, who guided the development of Florida’s anti-mosquito movement from the 1930s through the 1970s declared, “but it is going to take a greater job of informing the lawmakers, and other public officials, and the general public”. Mulrennan, on the eve of retirement, admonished his colleagues to maintain high standards. He observed:

“There is an old and simple adage ‘your sins will find you out.’ There have been occasions where local operations have not carried out recognized and approved mosquito control practices. Such instances have a detrimental effect on all mosquito control operations. There is a right and a wrong way to carry out an operation, but it seems, today there are those who feel they can carry out actions not recognized as legitimate, and in some instances may be over the borderline. Under these circumstances, the image of mosquito control is suspect and will be difficult to defend in the future [36] (p. 52).”

Maurice Provost, the director of Florida’s Entomological Research Center in Vero Beach, expressed his discontent with the over reliance on chemical means of control in even stronger terms. Provost declared:

“The day is gone when we can improve our relations with fellow man or fellow animals by simply reaching for a gun. We need to understand, and for this we must learn. And so we need only learn, learn, and learn some more to appreciate that our natural environment is full of wonder and beauty, even if we learn about some practical phenomenon as the common milieu of birds, mosquitoes, and viruses [37] (p. 5)”.

Provost and others believed that the proposal of four University of California entomologists (Vern Stern, Ray Smith, Robert van den Bosch, and Kenneth Hagen) for “The Integrated Control Concept” [38] (pp. 81–101) offered a viable alternative to the chemical era’s reliance on insecticides. This paper formed the basis of what later became known as integrated pest management (IPM) and integrated mosquito management (IMM). The key to making IMM a reality lay in research.

In California, Richard Frolli, manager of the Kings Mosquito Abatement District, echoed Provost’s concerns. “These are crucial times”, Frolli observed, “our pesticides are failing! Our basic solutions for mosquito control are dying! We must change our basic strategy, we must change our basic solution, we must change our district images to one other than spray districts if we are to be effective in mosquito abatement.” Like Provost, Frolli believed that the mosquito control movement had come to a turning point. “We have a crisis in mosquito control”, Frolli declared, “the crisis is a mental one—a reluctance to accept change. But this crisis will break as a fever does. Someday, we’ll all realize and the public as well, just how ridiculous the dependence upon repetitive spraying is as we get a clear grasp of the new realities of mosquito abatement, and define clearly new values and priorities [39] (pp. 1–2).”

Cutbacks in funding research on mosquitoes and mosquito control, however, were to become the norm in the closing decades of the twentieth century. On 6 June 1978, voters in California approved Proposition 13 (Prop 13) cutting property taxes by 57%. The draconian reduction in tax revenues carried both immediate and long term consequences for mosquito control. In northern California the revenues available to the Alameda County Mosquito Abatement District dropped by nearly 63% [40] (p. 15). Other California districts faced similar cuts.

The challenges facing mosquito control multiplied in the closing decades of the twentieth century. In Florida, state officials reorganized the government’s administrative units. The Florida State Board of Health (FSBH) and its entomology program became part of the Florida Department of Health and Rehabilitative Services (FDHRS). In 1974, James Bax, FDHRS’s head, instructed his division heads to focus their “efforts on providing service to individuals”. FDHRS transferred responsibility for the control of pesticides to the Florida Department of Agriculture and Consumer Services (FDACS). The FSBH’s Entomology Research Center, by then called the Florida Medical Entomology Laboratory (FMEL) became an “orphan” division [41] (p. 1).

Cutbacks in funding, the FMEL’s status as an “orphan” division, and the intense acrimony between Maurice Provost (FMEL’s director) and Jack Rogers, then head of the FDHRS entomology unit, reached a crisis point in 1979. Provost succeeded in winning legislative support for the transfer of FMEL to the University of Florida. The transfer took two years to complete. During this period, the tension between FDHRS’s leaders in Jacksonville and Tallahassee and the FMEL did not abate. In January 1979, the staff at FMEL distributed a detailed report, which summarized the efforts to “pressure [the researchers] to conduct broad advocacy research”. The report concluded: “It is not the intent of the Florida Medical Entomology Laboratory staff to embarrass any of the foregoing gentlemen but to demonstrate that the administrators of social programs or pursuers of limited objectives are inadequate for administering research. Nothing would be solved by the appointment of new administrators as their outlook could be little different.” Maurice Provost’s standing as an internationally renowned researcher had served to shield the laboratory from meddlesome bureaucrats. “Since Dr. Provost’s death”, the staff members declared, “only pending legislative bills to transfer FMEL to the University system have restrained hands that would reshape the reputation of the FMEL [42] (p. 6).” The transfer of FMEL to the University of Florida was completed in the early 1980s. Under the leadership, of the directors of FMEL, Richard Baker and followed by Walter Tabachnick the laboratory enhanced its reputation as one of the leading American medical entomological research facilities.

Critics of mosquito control have long claimed that the impounding of coastal salt marshes had damaged environmentally sensitive wetlands. In Florida, the management of these impoundments underwent significant improvement in the 1980s. In 1982, the U.S. Department of Commerce launched an eight year study of Coastal Zone Management (CZM) practices. In Florida, Jorge Rey (FMEL), Grant Gilmore (Harbor Branch Oceanographic Institution) and Doug Carlson (Indian River County Mosquito Control District) authored a detailed analysis of the of the effects of different water management strategies on coastal salt marshes [43]. They concluded that Rotational Impoundment Management (RIM) offered a means of protecting the environmentally sensitive wetlands while reducing pest mosquito populations. In 1989, Florida’s Subcommittee on Managed Marshes published a new set of guidelines that led to the widespread adoption of the RIM approach to coastal management.

It is beyond the scope of this chronicle to provide a detailed examination of the recent history of mosquito control. Three developments marked the character of the integrated era of mosquito control: the development of biopesticides; the arrival of invasive species; and, the threat posed by emerging pathogens. In 1975 A.S. Tahori and J. Margalit began a study of biological control agents. In August 1976, Margalit discovered the effectiveness of *Bacillus thuringiensis* var. *israelensis* (*Bti*) as a larvicide. The success of *Bti* led researchers to explore other bacterial agents. In the mid-1980s, Mir Mulla at the University of California, Riverside and others identified the efficacy of *Bacillus sphaericus* as a control agent [44] (p. 307). At present, considerable interest is being shown in the use of the bacteria *Wolbachia* as a biological control agent [45].

Increasing trade and movement of people, flora, and fauna that defines globalization have had a profound impact on mosquito control. Invasive mosquito species and emerging pathogens are at the center of contemporary research on mosquito control. In 1985, *Ae. albopictus*, “the Asian tiger mosquito”, was found in Harris County, Texas. In the next 20 years, this invasive species from Asia became established as one of the principal mosquito pests along the East Coast of the United States. In 2001, *Ae. albopictus* was found in shipments of “Lucky Bamboo” in the Port of Los Angeles [46]. Despite the concerted efforts of mosquito control workers, efforts to eradicate this species proved futile. Perhaps more disturbing was the discovery of *Ae. aegypti* in June 2013 outside of Fresno, California. Public health authorities are understandably concerned about these invasive species given the fact that both are competent vectors of a wide range of viruses.

The arrival of West Nile virus (WNV) in New York City in 1999, the first reported entry of this African virus into the United States, was a watershed moment in the history of mosquito control. During the next ten years WNV spread throughout the continental U.S. WNV revealed mosquito control’s strengths and weaknesses. Organized mosquito control launched campaigns against the *Culex* mosquitoes which transmitted the virus. In California “Fight the Bite” became the mantra for the control initiative. Leaders of the anti-mosquito movement were understandably surprised when vocal opposition to the control efforts led to protests by those with concerns about the impacts of mosquito control on the environment.

Predicting the future is an enterprise best left to psychics and astrologers. There is, however, reason to be hopeful about the future of mosquito control. Advances in genetic engineering and the work of Australian researchers such as Brian Kay, Richard Russell, and Scott O’Neill on the bacterium *Wolbachia* holds considerable promise for developing innovative means of preventing the spread of mosquito-borne diseases. In 2005, the Bill and Melinda Gates Foundation named O’Neill recipient of a Grand Challenge in Global Health Award for his proposal on “Modifying Mosquito Population Age Structure to Eliminate Dengue Transmission”. In the decade following the award O’Neill and his colleagues in the Eliminate Dengue Program [47] have made considerable progress. Undoubtedly, obstacles remain but the Australians’ work holds promise for the future of mosquito control.

The Australians’ efforts point to another critical element in the development of mosquito control: The task of building community support. At the beginning of the twentieth century, women played an important role in rallying support for “Suppress the Mosquito” campaigns. Winning local support for mosquito control is no less important today than it was 100 years ago. O’Neill and his colleagues’ projects in Cairns, Australia, Vietnam, Indonesia, Columbia, Venezuela, and Brazil demonstrate this. To succeed advocates of mosquito control must win the public’s support. O’Neill put it succinctly when he observed, “The project had gone beyond the science or managing a large laboratory. I had to develop new skills to communicate with government regulators, the people living in the communities, the media, [as well as] overseeing the field sites.” [48].

Finally, there have been significant advances in research on mosquito repellents. In the 60 years since the discovery of DEET, researchers have sharpened their understanding of mosquitoes’ olfactory system [49]. At the same time, new products such as picardin and natural products like oil of lemon eucalyptus are gaining popularity [50]. These advances hold promise both for the development of new means of protection and winning the public’s use of repellents.

The development of mosquito control in the 21st century will in large measure be determined by the success of control efforts in facing the challenge posed by the resurgence of dengue fever and the appearance of Zika virus on the world stage. *Aedes. aegypti* mosquitoes are the prime vector of both of these flaviviruses. There is as of yet no effective vaccine for either disease. Mosquito control and, in the case of Zika the practice of safe sex, remain the only means to break the transmission of these viruses. Unfortunately, though mosquito control has proven its effectiveness against many pest and pathogen-bearing mosquitoes, the recent experience with Zika has demonstrated that breaking the transmission cycle of mosquito-borne pathogens during an *Ae. aegypti*-borne epidemic remains at best problematical. Research on topics to improve mosquito control effectiveness continue with the development of new strategies for control including chemical, biological, genetically modified organisms, repellents, attractants for trapping and many more. History shows us that progress in mosquito control is dependent on the research efforts of dedicated scientists and mosquito control professionals.

Von Goethe’s contemporary, Georg Wilhelm Friedrich Hegel, once observed that “the only thing people have learned from history, is that no one ever learned anything from studying history”. The history of mosquito control and medical entomology provides some measure of optimism in the face of Hegel’s pessimism. Much has been learned about mosquitoes since von Goethe and Frederike’s encounter with the “entsetzlichen Rheinschnaken”. The research efforts of countless medical entomologists have deepened our understanding of the role of mosquitoes in transmitting pathogens that cause human diseases. If there is a lesson in the history of mosquito control, it is that there are no “magic bullets”. Protecting the public from pathogen bearing and nuisance mosquitoes depends on research to improve mosquito control and an abiding respect for the environment. When at its best, mosquito control is applied ecology.
